# Role of A-Kinase anchor protein (AKAP4) in growth and survival of ovarian cancer cells

**DOI:** 10.18632/oncotarget.18163

**Published:** 2017-05-24

**Authors:** Vikash Kumar, Nirmala Jagadish, Anil Suri

**Affiliations:** ^1^ Cancer Microarray, Genes and Proteins Laboratory, National Institute of Immunology, Aruna Asaf Ali Marg, 110067, New Delhi, India

**Keywords:** AKAP4, PKA, reactive oxygen species, s-phase arrest, apoptosis

## Abstract

Ovarian cancer represents one of the most common malignancies among women with very high mortality rate worldwide. A-kinase anchor protein 4 (AKAP4), a unique cancer testis (CT) antigen has been shown to be associated with various malignant properties of cancer cells. However, its involvement in various molecular pathways in ovarian cancer remains unknown. In present investigation, employing gene silencing approach, we examined the role of AKAP4 in cell cycle, apoptosis and epithelial-mesenchymal transition (EMT). Further, we also investigated the effect of ablation of AKAP4 on tumor growth in SCID mice ovarian cancer xenograft mouse model. Our results showed that ablation of AKAP4 resulted in increased reactive oxygen species (ROS) generation, DNA damage, cell cycle arrest and apoptosis in ovarian cancer cells. AKAP4 knockdown lead to degradation of protien kinase A (PKA) which was rescued by proteosome inhibitor MG-132. ROS quencher N-acetyl cysteine (NAC) treatment rescued cell cycle arrest and resumed cell division. Subsequently, increased expression of pro-apoptotic molecules and decreased expression of pro-survival/anti-apoptotic factors was observed. As a result of AKAP4 depletion, DNA damage response proteins p-γH2AX, p-ATM and p21 were upregulated. Also, knockdown of CREB resulted in similar findings. Further, PKA inhibitor (H89) and oxidative stress resulted in similar phenotype of ovarian cancer cells as observed in AKAP4 ablated cells. Collectively, for the first time our data showed the involvement of AKAP4 in PKA degradation and perturbed signaling through PKA-CREB axis in AKAP4 ablated ovarian cancer cells.

## INTRODUCTION

Ovarian cancer is a complex disease with very high mortality rate among all gynecological cancers [1]. Majority of patients have recurrent ovarian cancer that occurs shortly after chemotherapy with limited available treatment options [2]. Therefore, there is an urgent need to develop novel therapeutic treatment for better cancer management. In this regard, a unique class of tumor associated antigens known as cancer testis (CT) antigens have been shown to be associated with different types of cancer [3]. Recently, role of CT antigens has been demonstrated in various malignant properties of cancer cells [4]. In addition, CT antigens are highly immunogenic and have tumor restricted expression and hence are now being explored as cancer therapeutic vaccine [5].

Earlier, we reported a novel CT antigen namely A kinase anchor protein (AKAP4) expression in various types of malignancies [6–9]. It has been proposed that AKAP4 functions as a scaffolding protein and tethers protein kinase A (PKA) in various signaling complexes and subcellular organelles [10]. PKA regulates various aspects of physiology including cell growth, motility and apoptosis [11]. PKA phosphorylates and mediates its effects through various substrate including cyclic AMP response element binding protein (CREB), a known regulator of various oncogenic processes in the cell [11, 12]. In this context, pro-survival nature of PKA-CREB signaling has been reported in NIH3T3 k-ras transformed cells under glucose starvation [13] and maintenance of ROS homeostasis in melanocytes [14]. In addition, PKA anchoring and its role in metastasis has been proposed in ovarian cancer cells [15].

Recently, in colorectal cancer cell line model, we showed that ablation of AKAP4 lead to cell cycle arrest, cell death, inhibited migration, invasion and reduced tumor growth [16]. Yet another study showed that ablation of AKAP4 resulted in reduced tumor growth in esophageal cancer xenograft mice model [17]. However, the putative function and involvement of AKAP4 in various signaling modules and in PKA-CREB signaling has not been studied in ovarian cancer cells so far. Therefore, in the present study, employing gene silencing approach, we investigated the effect of ablation of AKAP4 in ovarian cancer cells which showed decreased expression of PKA and CREB. Further, various signaling cascades and malignant properties involving PKA and CREB were investigated and found compromised after AKAP4 ablation. Our data suggests that AKAP4 may play an important role for ovarian cancer cell survival, and it may be targeted as a novel therapeutic intervention in ovarian cancer.

## RESULTS

### AKAP4 gene, protein expression and FACS

**Figure 1:**
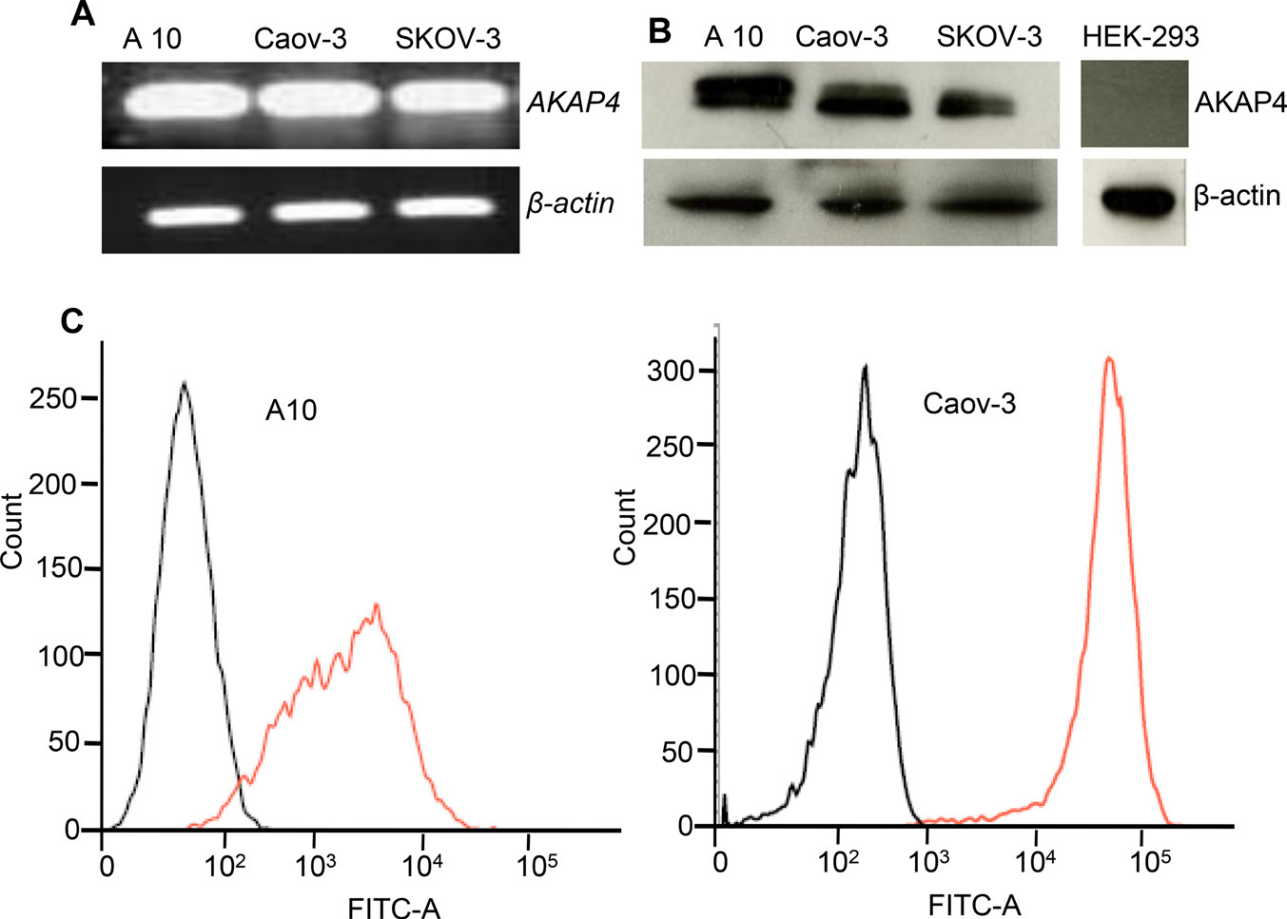
AKAP4 gene, protein expression and surface localization (**A**) RT-PCR shows *AKAP4 gene* expression in ovarian cancer cell line A10, Caov-3 and SKOV3. (**B**) Western blot shows AKAP4 protein expression in A10, Caov-3, SKOV3 and HEK-293 (negative control). β- actin serves as loading control. (**C**) FACS analysis shows surface expression of AKAP4 protein in A10 and Caov-3. FITC positive cells are shown on X-axis in histogram overlay, which shows AKPA4 expression (orange line) in A10 (98%) and Caov-3 (99%) verses (6%) and (4%) in unstained population (black line) of A10 and Caov-3 respectively. The data shown as mean ± standard error of the mean (SEM) of three independent experiments. *P < 0.05; **P < 0.01.

*AKAP4* gene expression was examined by RT-PCR which showed presence of *AKAP4* gene expression in all three ovarian cancer cells (Figure 1A). Further, gene expression was validated by Western blotting which showed AKAP4 protein expression (Figure 1B). AKAP4 expression is not seen in HEK-293. Subsequently, AKAP4 surface localization was evaluated by fluorescent activated cell sorting (FACS), which revealed 98% in A10 cells and 99% in Coav-3 cells surface localization as compare to 6% and 4% in unstained A10 and Coav-3 cells (Figure 1C).

### AKAP4 knockdown inhibits cellular proliferation and cell viability

**Figure 2:**
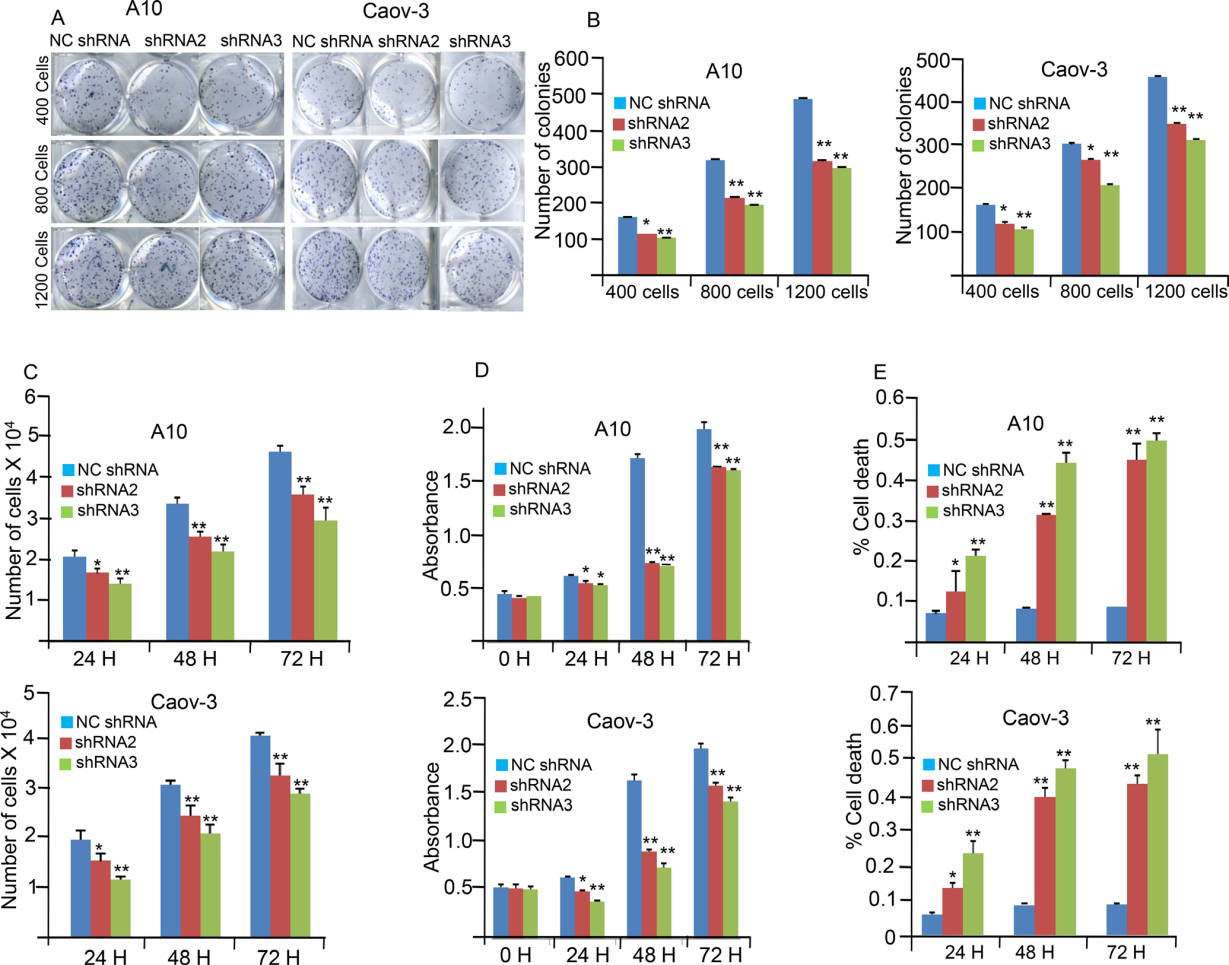
AKAP4 knockdown inhibits colony forming ability, cellular proliferation and cell viability (**A** and **B**) Image and Bar diagram shows colony formation ability of A10 and Caov-3 after NC shRNA, shRNA2 and shRNA3 treatment. Significant inhibition in colony forming ability was observed in shRNA2 and shRNA3 treated cells compare to NC shRNA treated cells (**C**) Bar diagram depicts reduced cellular proliferation at 24 h, 48 h and 72 h in A10 and Caov-3 after AKAP4 knockdown. (**D**) Bar diagram depicts MTT assay at 0 h, 24 h, 48 h and 72 h after NC shRNA, shRNA2 and shRNA3 treatment. (**E**) Trypan blue cell exclusion assay shown in the bar diagram with significant increase in cell death after AKAP4 ablation. The data shown as mean ± standard error of the mean (SEM) of three independent experiments. *P < 0.05; **P < 0.01.

Effects of AKAP4 ablation on various malignant properties of cancer cells were investigated in A10 and Caov-3 cells. Cellular proliferation was significantly inhibited in shRNA2 treated (*P* = 0.003 and *P* = 0.006) and shRNA3 treated (*P* = 0.0001 and *P* = 0.0008) in A10 and Caov-3 cells respectively (Figure 2C) compared to NC shRNA treated A10 and Caov-3 cells. Colony forming ability was also investigated and found significantly inhibited in shRNA2 treated (*P* = 0.001) and shRNA3 treated (*P* = 0.0001; Figure 2A and 2B) as compared to NC shRNA treated A10 and Caov-3 cells. Further, effect of AKAP4 knockdown on cell viability was assessed by MTT (3-(4, 5- dimethylthiazolyl-2)-2, 5-diphenyltetrazolium bromide) assay in A10 and Caov-3 cells, which showed (Figure 2D) significant decrease in cell viability after shRNA2 (*P* = 0.0001and *P* = 0.004) and shRNA3 (*P* = 0.0001 and 0.003) treatment in A10 and Caov-3 cells respectively compared to NC shRNA treated cells. In addition, cell viability was also confirmed by Trypan blue exclusion method, which showed (Figure 2E) significant increase in non viable cell population after shRNA2 treatment (*P* = 0.006 and *P* = 0.004) and shRNA3 treatment (*P* = 0.007 and *P* = 0.005) in A10 and Caov-3 cells respectively, compared to NC shRNA treatment.

### AKAP4 knockdown induces cell cycle arrest

**Figure 3:**
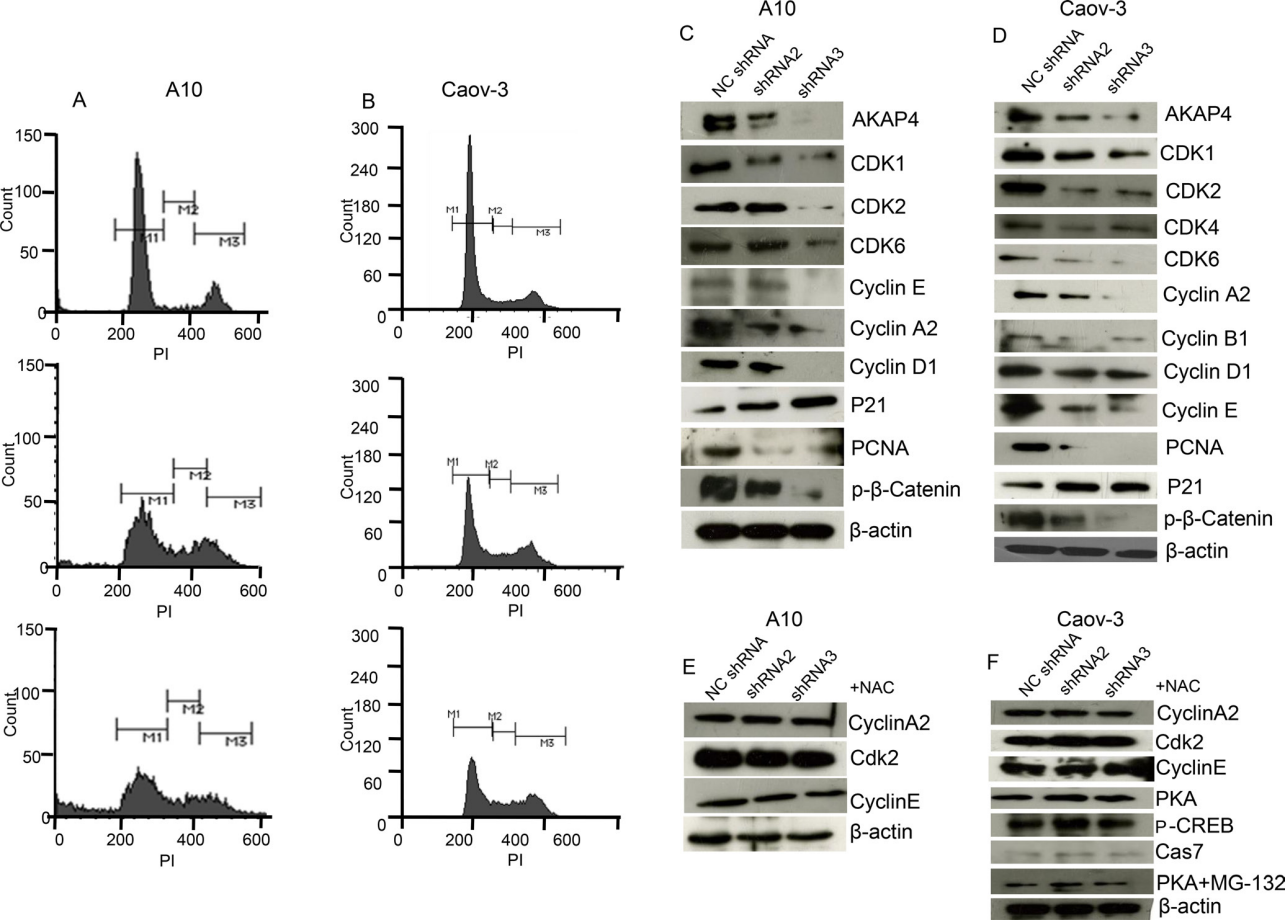
AKAP4 knockdown induces cell cycle arrest (**A** and **B**) Histogram shows s-phase growth arrest after PI staining at 48 h in A10 cells and Caov-3 cells respectively. (**C** and **D**) Western blot shows various cell cycle related proteins. (**E** and **F**) Western blot shows s-phase specific proteins after 4 mm NAC pre-treatment in A10 and Caov-3 cells and PKA, p-CREB and cleaved caspage7 in Caov-3. PKA rescue in Caov-3 is shown after MG-132 (500 nm for 1 h) pre- treatment, β- actin serves as loading control. The data shown as mean ± standard error of the mean (SEM) of three independent experiments. *P < 0.05; **P < 0.01.

Further to examine specifically at what phase of cell cycle, cancer cells are arrested post shRNA treatment, propidium iodide (PI) staining was performed. PI staining revealed s-phase arrest (Figure 3A and 3B) in shRNA2 (16%) and shRNA3 (21%) treated A10 cells and (13%) and (18%) in Caov-3 cells respectively as compared to (5%) in A10 and (7%) in Caov-3 NC shRNA treated cells. We subsequently examined the expression of cell cycle related proteins by Western blotting (Figure 3C and 3D) and found decreased level of s-phase specific proteins cyclin A2, cyclin E and CDK2 which are important in terms of its regulation and progression. We also found decreased expression of CDK1, CDK4, CDK6, cyclin D1 and cyclin B1, indicating global inhibition in survival and cell cycle progression. Further we found decreased expression of PCNA and β-catenin, and increased expression of p21 tumor suppressor protein. Next we pre-treated ovarian cancer cells with NAC, a reactive oxygen species quencher to rescue cell cycle arrest. Interestingly, NAC (4 mm) pre-treatment recovered cells from s-phase cell cycle arrest and showed an increase of s-phase cell population by (3%) in case of shRNA2 + NAC treatment and (1%) in case of shRNA3 + NAC treatment compared to NC shRNA treated A10 cells (Supplementary Figure 1A). Similarly NAC pre-treatment in Caov-3 cells showed only (1%) increase in s-phase cell population in both shRNA2 + NAC and shRNA3 + NAC treatment compared to NC shRNA treated cells (Supplementary Figure 1A). Western blot was also carried out (Figure 3E and 3F) and no difference was found in the expression of s-phase specific proteins Cyclin A2, Cyclin E and CDK2 in A10 and Caov-3 and PKA, p-CREB and cleaved caspage7 in Caov-3. Further, mode of degradation of PKA was investigated by inhibiting proteosome mediated degradation. Pre-treatment (before shRNA treatment) with 500 nm MG-132 (proteosome inhibitor) for 1h blocked PKA degradation completely (Figure 3F and Supplementary Figure 1B), as shown in Western blot analysis in Caov-3.

### AKAP4 ablation leads to increased ROS generation DNA damage and cell death

**Figure 4:**
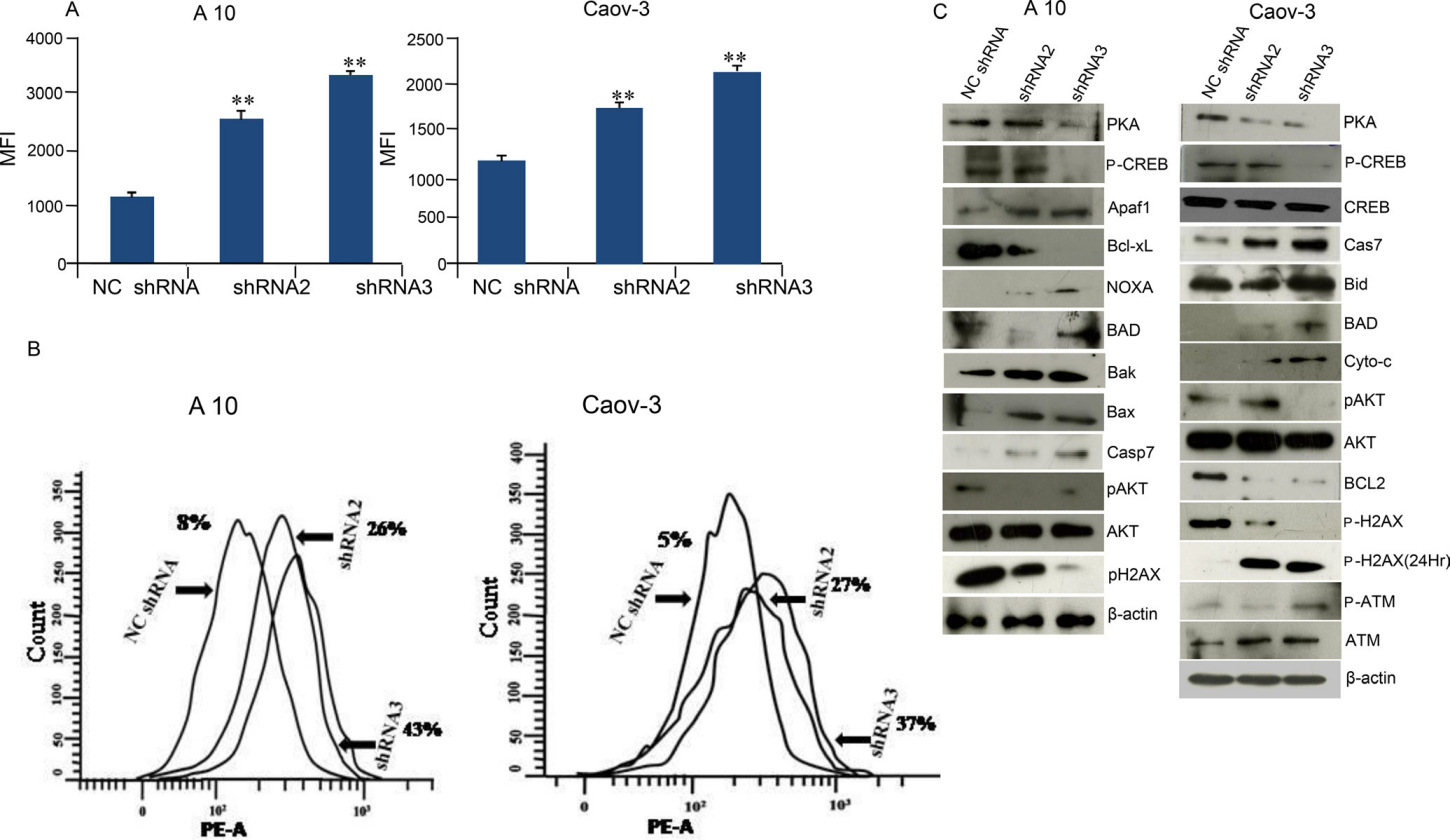
AKAP4 ablation leads to increased ROS generation DNA damage and cell death (**A**) Bar diagram represents shows significant increase in ROS generation in MFI (mean fluorescence intensity) after NC shRNA, shRNA2 and shRNA3 treatment in A10 and Caov-3. (**B**) Histogram shows increased DNA damage through TUNEL assay after NC shRNA, shRNA2 and shRNA3 treatment in A10 and Caov-3 cells. (**C**) Western blot in A10 and Caov-3 cells show upregulation of pro-apoptotic proteins and downregulation of pro-survival and anti-apoptotic proteins after NC shRNA, shRNA2 and shRNA3 treatment, β- actin serves as loading control. The data shown as mean ± standard error of the mean (SEM) of three independent experiments. *P < 0.05; **P < 0.01.

Intracellular ROS generation after shRNA treatment in A10 and Caov-3 cells was evaluated by CellRox Deep Red reagent. As shown in Figure 4A, cells treated with shRNA2 in A10 and Caov-3 showed 2.39 fold and 1.5 fold increase in ROS generation respectively. Similar results were obtained when cells were treated with shRNA3 which showed 2.77 fold and 1.73 fold increased ROS generation respectively compared to NCshRNA treated cells. In order to investigate the gene silencing effect of AKAP4 knockdown on DNA fragmentation and apoptosis, TUNEL (terminal dUDP nick-end labeling) assay was performed in A10 and Caov-3 cells 48hr post transfection. TUNEL assay revealed increased BrdU positive cells (Figure 4B) in shRNA2 (26%) and shRNA3 (43%) in A10 cells and (27%) and (37%) in Caov-3 cells respectively as compared to (8%) in A10 and to (5%) in Caov-3 cells treated with NC shRNA. Further, specific proteins related to DNA damage response was evaluated by Western blotting. Phosphorylated γH2AX (H2A histone family, member X) in A10 and Caov-3 cells was found downregulated after AKAP4 ablation at 48 hr. However, p- γH2AX was found upregulated at 24 h and p-ATM which is an another marker of DNA damage was also found elevated at 48h (Figure 4C). Further, to confirm caspase mediated cell death in AKAP4 ablated cells, Western blot revealed (Figure 4C) increased expression of pro-apoptotic molecules Apaf-1, BAD, Bak, Bax, and cleaved caspase 7 in A10 cells and Bid, BAD, Cyto-c and cleaved caspase 7 in Caov-3 cells. Decreased expression of anti- apoptotic proteins Bcl-xL in A10 and Bcl-2 in Caov-3 cells was also observed. Interestingly, caspase inhibitor rescued apoptotic phenotype and resumed cell division as shown in PI staining (Supplementary Figure 2A). In addition, no difference was seen in PCNA, PAPR1, p-ATM (Supplementary Figure 2B). To evaluate whether AKAP4 knockdown has any effect on PKA mediated signaling, we carried out Western blotting and found decreased expression of PKA catalytic subunit in A10, Caov-3 and SKOV-3 cells (Figure 4C and Supplementary Figure 1C) post shRNA2 and shRNA3 treatment compare to NC shRNA treated cells. Phosphorylation of CREB was also shown to be downregulated after AKAP4 knockdown. Another important regulator of cell growth and metabolism is AKT [18], which was also found to be downregulated after AKAP4 ablation in A10 and Caov-3 cells (Figure 4C).

### AKAP4 knockdown inhibited wound healing ability and metastatic markers in A10 and Caov-3 cells

**Figure 5:**
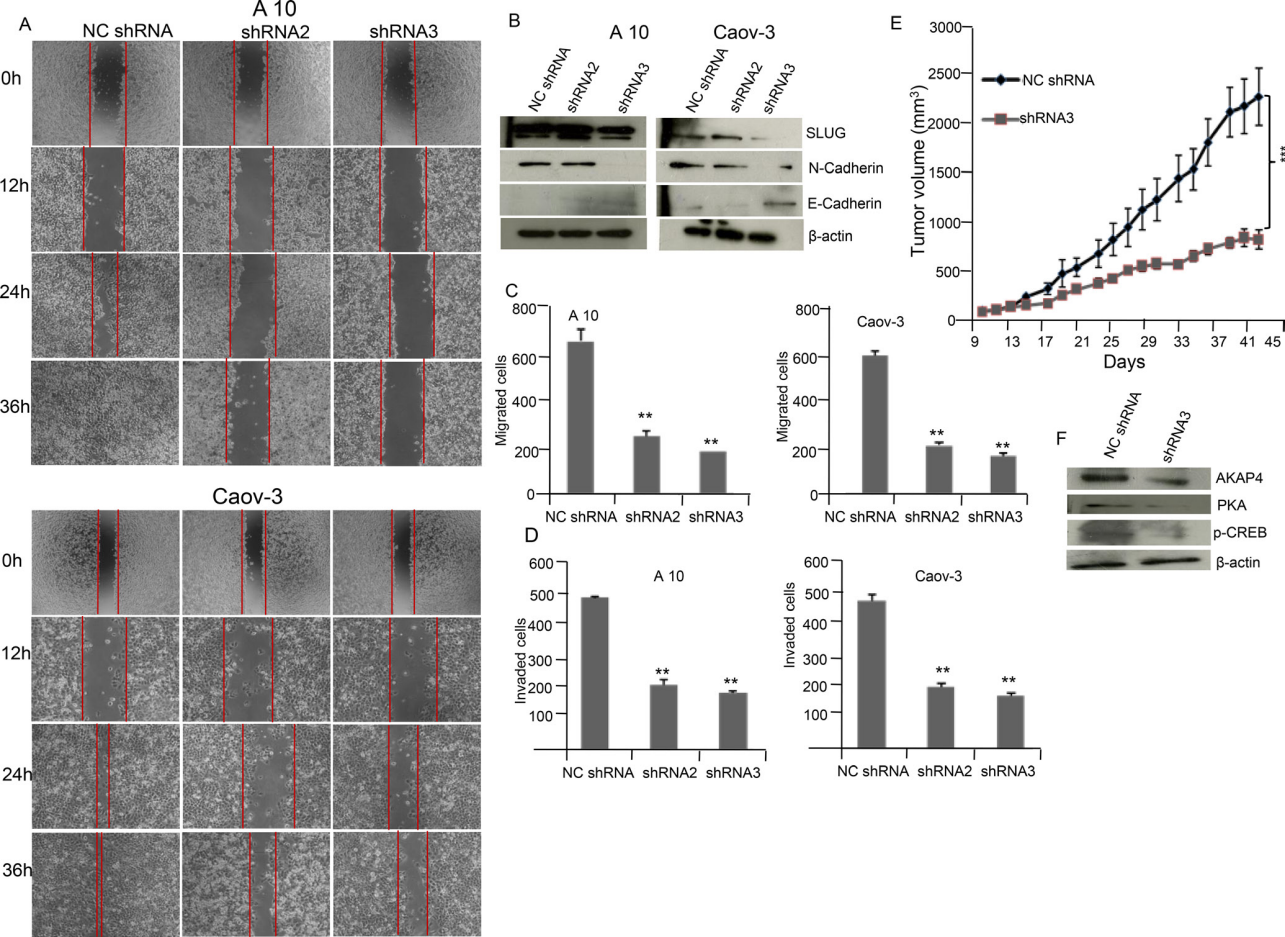
AKAP4 knockdown inhibits wound healing ability and cellular motility marker in A10 and Caov-3 cells and reduces tumor growth in SCID mice model (**A**) Phase contrast microscopy shows decreased wound healing ability in A10 and Caov-3 cells after NC shRNA, shRNA2 and shRNA3 treatment. (**B**) Western blot shows decreased expression of mesenchymal marker and increased expression of epithelial marker in A10 and Caov-3, β- actin serves as loading control. (**C** and **D**) Bar diagram shows significant reduction in migrated and invaded cells after AKAP4 ablation in A10 and Caov-3 cells compared to NC shRNA treated cells. (**E**) Tumor volume was reduced after shRNA3 treatment significantly (*P* = 0.001) in SCID mice model compared to NC shRNA treated mice. (**F**) Western blot shows decreased expression of AKAP4, PKA and p-CREB in shRNA3 treated tumor lyaste. β- actin serves as loading control. The data shown as mean ± standard error of the mean (SEM) of two independent experiments. *P < 0.05; * * *P* < 0.01, * * * *P* < 0.001.

In order to study the migration ability phase contrast images (Figure 5A) were acquired in A10 and Caov-3 cells post NC shRNA, shRNA2 and shRNA3 treatment. Significant reduction in wound healing ability was observed in shRNA2 treated (*P* = 0.001) and shRNA3 treated (*P* = 0.0001) cells compared to NC shRNA cells in both A10 and Caov-3 cells. The wound was completely healed in NC shRNA treated A10 cells within 36 h but not in shRNA2 and shRNA3 treated A10 cells. Further, Western blot analysis (Figure 5B) supported decreased metastatic potential revealing decreased expression of mesenchymal marker N-cadherin and SLUG and increased expression of epithelial marker E-cadherin in A10 and Caov-3 cells after AKAP4 ablation by shRNA2 and shRNA3 treatment compared to NC shRNA. Migration assay showed 62% and 73% reduction in A10 and 64% and 66% in Caov-3 after shRNA2 and shRNA3 treatment (Figure 5C). Similarly, Invasion assay showed 58% and 63% reduction in A10 and 59% and 64% in Caov-3 after shRNA2 and shRNA3 treatment compared to NC shRNA treated cells (Figure 5D).

### AKAP4 ablation in ovarian cancer xenograft reduces tumor growth

Effect of ablation of AKAP4 on tumor growth in SCID mice ovarian cancer xenograft model was evaluated. Intra-tumor injection of 50 μg NC shRNA or shRNA3 was given to control and experimental group on alternate days. Significant reduction (*P* = 0.001) in tumor volume was observed in *in vivo* tumor xenograft (Figure 5E) after shRNA3 treatment as compared to NC shRNA treatment. Western blot of tumor lysate showed (Figure 5F) decreased expression of AKAP4, PKA and p-CREB in shRNA3 treated animals compared to NC shRNA animals.

### H89 and ROS treatment resulted in similar phenotype of Caov-3 cells

**Figure 6:**
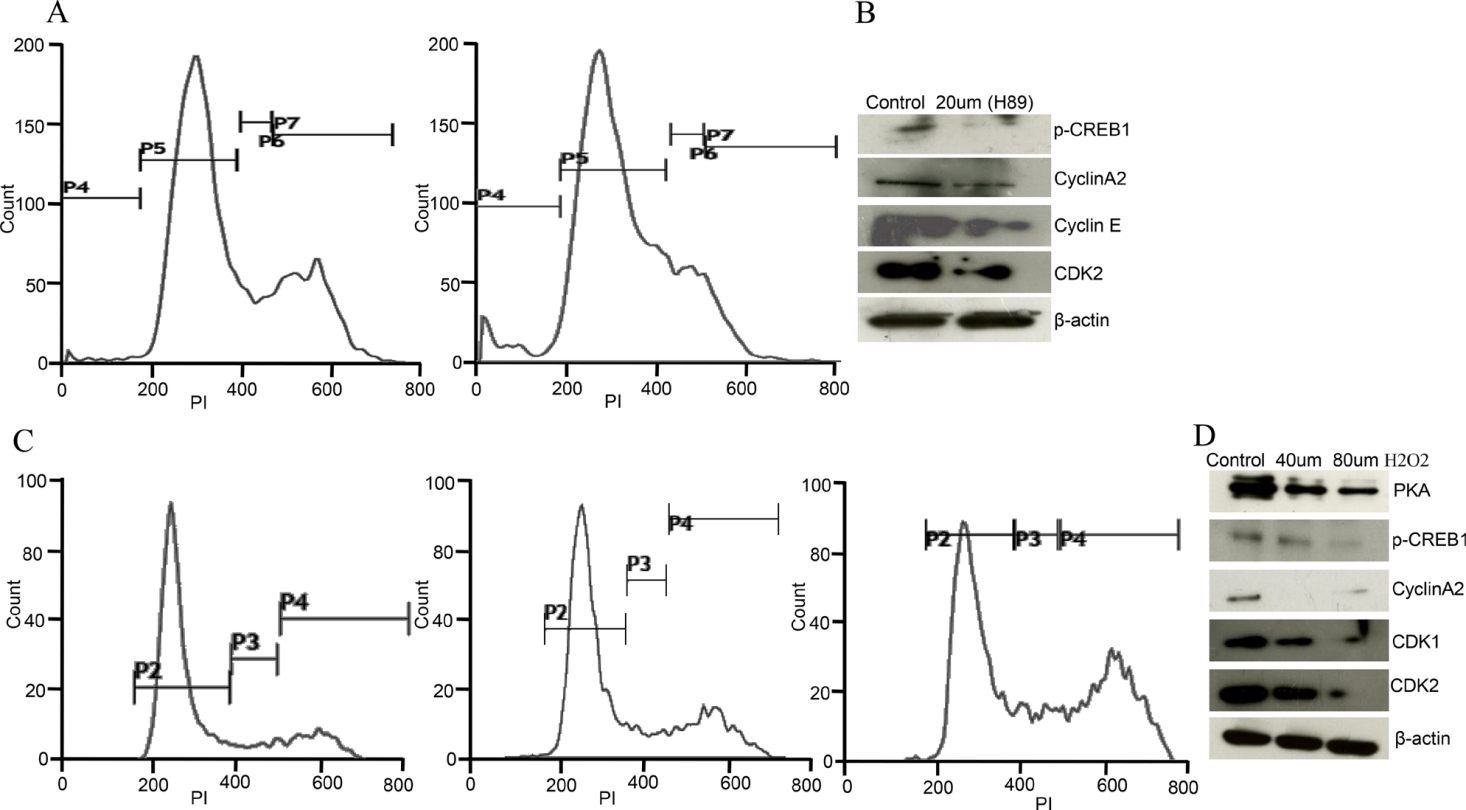
H89 and ROS treatment show similar phenotype in Caov-3 cells (**A**) Histogram shows s-phase cell cycle arrest after PI staining treated with H89 (30 μm) in Caov-3 cells. (**B**) Western blot shows downregulated p-CREB, cyclinA2, cyclinE, and CDK2 after 30 μm H89 treatment in Caov-3 cells. (**C**) Representative histogram analysis shows S/G2M cell cycle arrest after H2O2 treatment (untreated, 40 μm and 80 μm) for 24 h in Caov-3 cells. (**D**) Western blot analysis shows downregulated PKA, p-CREB, cyclinA2, CDK1 and CDK2 after 40 μm and 80 μm H2O2 treatment in Caov-3 cells, β- actin serves as loading control. The data shown as mean ± standard error of the mean (SEM) of three independent experiments. *P < 0.05; **P < 0.01.

PKA inhibitor (H89) and oxidative stress (H2O2 treatment) resulted in similar phenotype of ovarian cancer cells as observed in AKAP4 ablated cells. To evaluate direct impact of PKA inhibition, PI staining was carried out after H89 treatment in Caov-3 cells, which showed (Figure 6A) accumulation of 13% cells in s-phase after (30 μm H89) treatment compared to 6% cells in s- phase in untreated cell population. An increase in sub G0/G1 population and decrease in G2/M- phase cells was also observed after H89 treatment. Western blot analysis revealed (Figure 6B) decreased expression of p-CREB, cyclin A2, cyclin E and CDK2 post H89 treatment, confirming s-phase cell cycle arrest. Similarly, direct impact of ROS on cell cycle was also evaluated after H2O2 treatment. PI staining was carried out, which showed accumulation of 9% cells in s-phase and 23% cells in G2/M-phase when treated with 40 μm H2O2 in Caov-3 cells and 13% cells in s-phase and 31% cells in G2/M-phase when treated with 80 μm H2O2 compared to 8% cells in s-phase and 19% cells in G2/M-phase in untreated population at 24h (Figure 6C). Western blot analysis further supported cell cycle arrest and showed (Figure 6D) decreased expression of PKA, p-CREB, cyclin A2, CDK1 and CDK2 after H2O2 treatment in Caov-3 cells.

### CREB knockdown mimicked AKAP4 ablated phenotype in ovarian cancer cells

**Figure 7:**
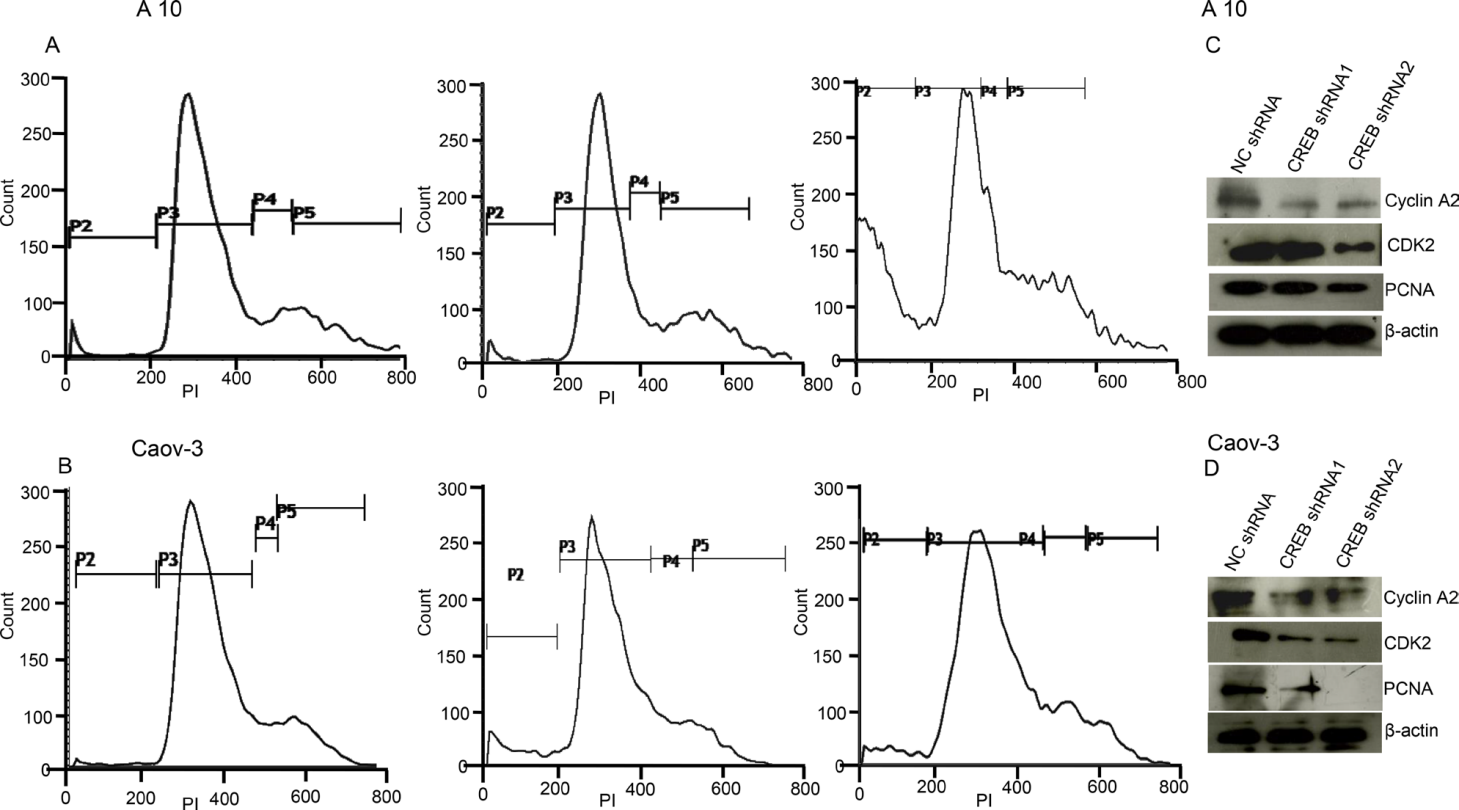
CREB knockdown mimics AKAP4 ablated phenotype in A10 and Caov-3 cells (**A** and **B**) Histogram shows s-phase cell cycle arrest after CREB ablation in A10 and Caov-3 cell after PI staining at 48 hr. (**C** and **D**) Western blot analysis shows s-phase specific molecules along with PCNA. β- actin serves as loading control. The data shown as mean ± standard error of the mean (SEM) of three independent experiments. *P < 0.05; **P < 0.01.

**Figure 8:**
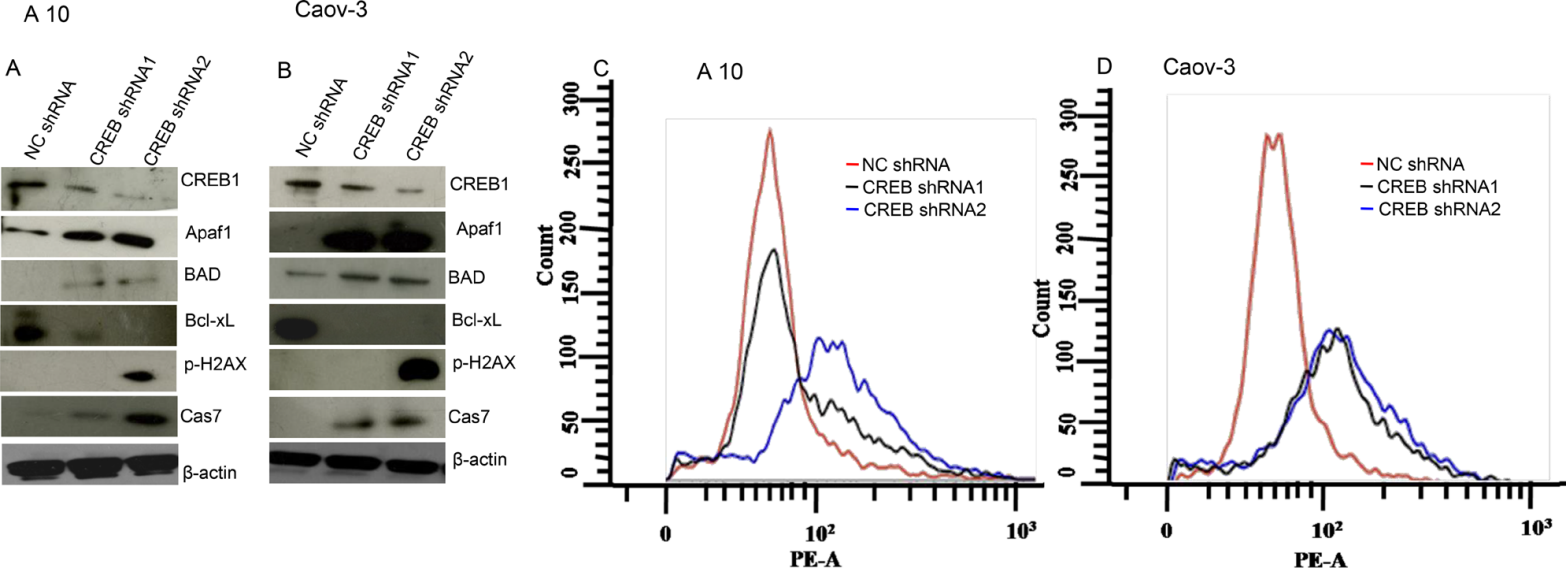
CREB knockdown induces DNA damage and cell death in A10 and Caov-3 cells (**A** and **B**) Western blot shows upregulation of pro-apoptotic proteins and downregulation of anti-apoptotic protein after CREB ablation in A10 and Caov-3 cells. β- actin serves as loading control. (**C** and **D**) TUNEL assay shows increased DNA damage after CREB ablation. The data shown as mean ± standard error of the mean (SEM) of three independent experiments. *P < 0.05; * * *P* < 0.01.

Ablation of CREB in ovarian cancer cells (A10 and Caov-3) mimicked AKAP4 ablated phenotype. PI staining after CREB ablation showed s-phase growth arrest in A10 and Caov-3 cells. In A10, shRNA1 and shRNA2 treatment showed (9%) and (13%) cells in s-phase, compared to 8% cells after NC shRNA treatment (Figure 7A). Similarly, CREB ablation in Caov-3 cells showed (13%) and (12%) accumulation in s-phase after shRNA1 and shRNA2 treatment, compared to (6%) NC shRNA treated cells (Figure 7B). An increase in sub G0/G1 population was also observed in both the cell lines after CREB ablation. S-phase specific molecules (cyclinA2, CDK2 and PCNA) were found downregulated as shown by western blotting in shRNA1 and shRNA2 treated cells (Figure 7C and 7D). After CREB knockdown, expression of apoptosis associated molecules (Apaf-1, BAD and cleaved caspase 7) were found upregulated in ovarian cancer cells as compared to NC shRNA treated cells (Figure 8A and 8B). In addition, γH2AX, a known marker of DNA damage was found upregulated in A10 and Caov-3 cells after CREB ablation. TUNEL assay was performed in A10 and Caov-3 cells 48hr post transfection to examine DNA damage which revealed increase in BrdU positive cells (43% and 55%) after shRNA2 and shRNA3 treatment in A10 and (16% and 17%) in Caov-3 cells respectively, as compared to (7%) and 8%) in A10 and Caov-3 cells treated with NC shRNA (Figure 8C and 8D).

## DISCUSSION

Epithelial ovarian cancer (EOC) is one of the major gynecological cancers leading to cancer related death worldwide [1, 19]. Majority of EOC are diagnosed at advanced stage with poor prognosis and high morbidity [1]. Earlier we reported a novel molecule A kinase anchor protein 4 (AKAP4) [20] and showed its association with variety of malignancies [6–9]. In present study we investigated the role of AKAP4 in various malignant properties in ovarian cancer cells *in vitro* and in *in vivo* ovarian cancer xenograft mouse model. By gene silencing approach, ablation of AKAP4 in ovarian cancer cells provoked cell cycle arrest with decreased expression of key molecules involved in the various events of cell cycle and reduced tumor growth. In addition, onset of apoptosis was also observed in AKAP4 ablated cells due to increased expression of various pro-apoptotic molecules and decreased expression of anti-apoptotic proteins indicating that AKAP4 may be a new therapeutic target for cancer treatment.

In recent studies, putative function of AKAP4 in anchoring Protein kinase A (PKA) in facilitating downstream signaling involving various cellular compartments has been documented [10]. In addition, dysregulation of PKA signaling has been found in various types of cancers [11]. Also, PKA has been shown to regulate various physiological processes including cell growth, metabolism and cell differentiation [10]. It has been well documented that PKA mediates its downstream effect through various substrates including cyclic AMP response element binding protein (CREB), which is a known regulator of multiple proto-oncogenes [12]. Interestingly, our study in AKAP4 ablated ovarian cancer cells showed decreased expression of p-CREB, β-catenin, PCNA, cyclinA2, cyclinB1, cyclinD1, cyclinE along with their CDK partners which has important role in cellular proliferation. This phenotype may be attributed due to decreased expression of p-CREB which has been shown to be involved in transcribing various cell cycle related proteins such as cyclinA2, cyclinD1 and PCNA [21–23].

Further, shRNA mediated knockdown of CREB resulted in similar phenotype. Interestingly, our data suggests that may be in EOC cells, AKAP4 mediated anchoring of PKA is important for cell cycle regulation and its progression which warrants further studies.

Intrigued by decreased expression of PKA due to AKAP4 knockdown, we further examined whether pharmacological inhibition of PKA could mimic AKAP4 ablated phenotype in ovarian cancer cells. Interestingly, pharmacological inhibition of PKA mimicked AKAP4 ablated phenotype in Caov-3 cells. Earlier studies have shown that pharmacological inhibition of PKA lead to increased reactive oxygen species (ROS) production [14], and its quenching by N-acetyl cysteine (NAC) reduced tumor progression through reduced genomic instability [24]. In this context, we examined whether NAC treatment could reverse the phenotype of ovarian cancer cells. As expected, cell cycle arrest was rescued after NAC treatment as suggested by our PI staining profile of NAC treated A10 and Caov-3 cells and Western blot analysis of s-phase specific proteins cyclin A2, cyclin E and CDK2. Further involvement of PKA degradation in AKAP4 depleted cells was examined using MG-132 proteosomal inhibitor which lead to complete blockade of PKA degradation, indicating proteosome mediated degradation of PKA in AKAP4 ablated cells. Our study, for the first time highlights a potential role of AKAP4 in proteosome mediated degradation of PKA and its consequences.

Diminished and inhibited apoptosis is an essential requirement for tumor formation and cancer progression [25]. The desired goal of most of the anti-cancer drugs is to trigger and accelerate the rate of apoptosis [26]. Earlier studies from our group and others laboratories have shown that knockdown of cancer testis (CT) antigens facilitated the process of apoptosis [16, 27]. However, associated mechanistic details and trigger of death pathways have not been studied so far. In present investigation, our data revealed that ROS may be a trigger and initiator, leading to caspase activation and cell death in AKAP4 ablated cancer cells. This is in line with an earlier study wherein it has been shown that cancer cells can be selectively targeted and may initiate cell death through increased ROS generation [28]. Further, in support of our finding it has been proposed that increased ROS generation may be responsible for DNA damage, cell cycle arrest and caspase activation in SKOV-3 cancer cells [29]. Earlier study has also shown the sensitivity of ovarian cancer cells towards high ROS leading to DNA damage as marked by increased phosphorylation of γH2AX (H2A histone family, member X) [30]. However, we found decreased phosphorylation of γH2AX in AKAP4 ablated cells at 48hr but increased at 24hr time point that suggests functions other than DNA repair and warrants further investigation. Moreover, increased DNA nicking as shown by TUNEL assay and increased expression of p-ATM is suggestive of DNA damage response and its associated consequences after AKAP4 ablation. Interestingly, caspase inhibition rescued the apoptosis, and resumed cell division. ROS is two-edged sword which cancer cells exploit very efficiently for growth and proliferation. Moderate level of ROS is required by cancer cells to divide and proliferate but excess of it can activate several death pathways [31]. Many known anticancer drugs like taxol, cisplatin, doxorubicin, curcumin are known to cause cell death through ROS generation [32, 33]. Here, we show increased ROS generation, DNA damage and subsequent caspase activation which lead to cell death after AKAP4 ablation and further studies are warranted to further explore the mechanistic detail of ROS and oxidative stress maintenance through PKA-CREB axis.

**Figure 9:**
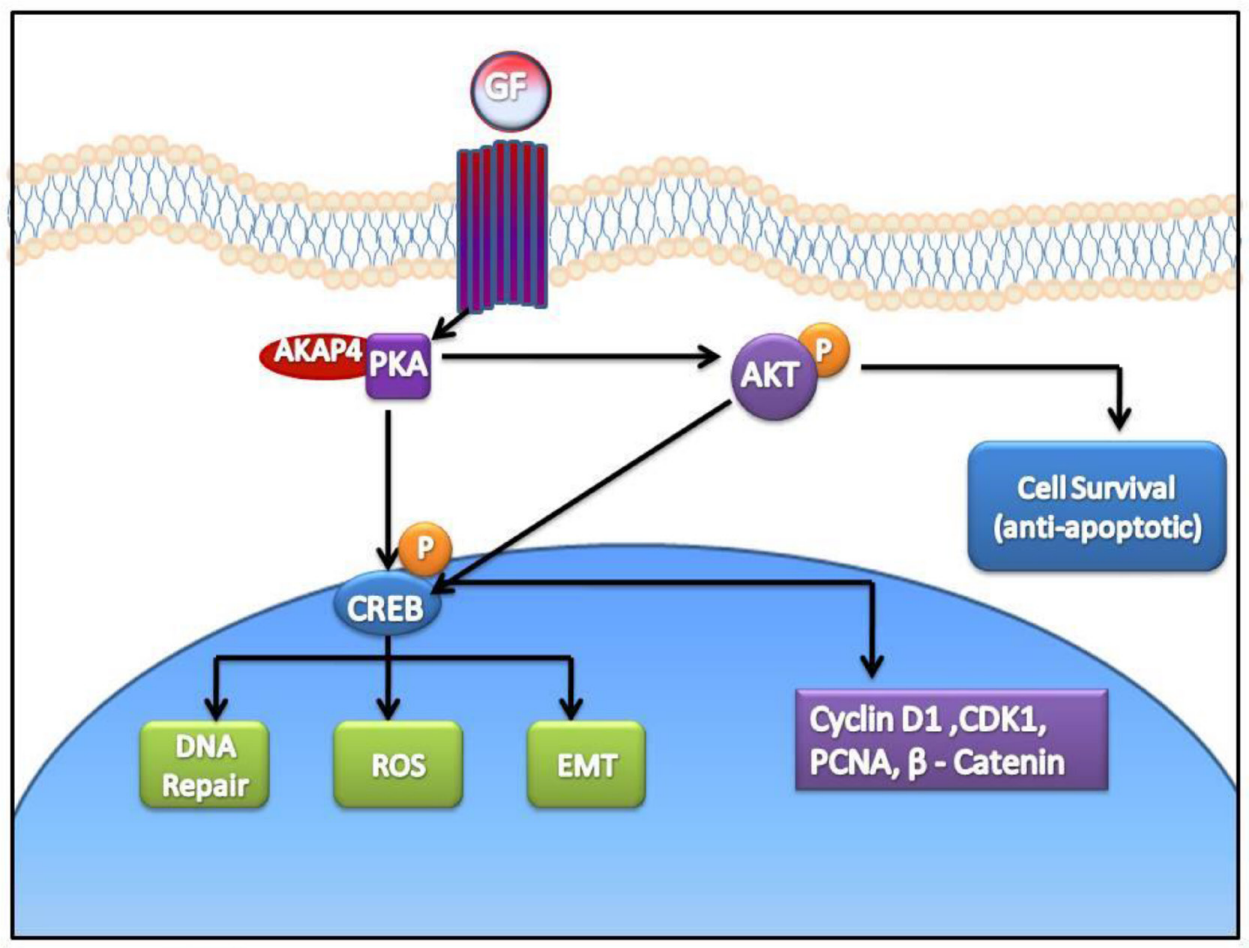
Cartoon representing anti- cancer effect of AKAP4 knockdown in ovarian cancer cells Ablation of AKAP4 lead to proteosome mediated degradation of PKA and consequently perturbed signaling through p-CREB. Various molecules involved in cell cycle regulation, apoptosis and metastasis were found deregulated after AKAP4 knockdown.

Yet, another aspect of ovarian cancer cell survival through increased phosphorylation of AKT has been well documented [18]. Similarly, we also found that AKAP4 ablation lead to the inhibition of pro-survival signal through decreased phosphorylation of AKT. Our study supported an earlier finding wherein it has been shown that PKA mediated AKT phosphorylation through follicular stimulating hormone (FSH) signaling is required for proliferation and survival of ovarian granulosa cells [34]. Clearly, our study shows that AKAP4 ablation lead to compromised survival of ovarian cancer cells through inhibition of various pro- survival signals. Thus, it is reasonable to state that ablation of AKAP4 lead to proteosome mediated degradation of PKA resulting in perturbed signaling through p-CREB (Figure 9).

Metastasis and drug resistance remains the major problem in cancer related deaths among cancer patients [35]. We investigated whether AKAP4 ablated cells have any impact on cell migration and invasion. We found reduced migration and invasion potential of AKAP4 depleted cells with inhibited expression of putative mesenchymal markers N-cadherin and SLUG and increased expression of E-cadherin. Interestingly, a recent study by Li et al has shown that AKAP4 overexpression can increase EMT in *in vitro* model [17]. PKA anchoring has shown to be involved in ovarian cancer cell migration and invasion leading to increased metastasis [15]. This is line with our data wherein, phenotypically AKAP4 depleted ovarian cancer cells had reduced invasive properties. However, mechanism of AKAP4 anchored PKA signaling favoring acquisition of metastatic behavior of cancer cells still requires further studies to provide better insight.

In conclusion our study suggested that AKAP4 might be playing role in ovarian cancer growth. Gene silencing approach revealed that various oncogenic properties of ovarian cancer cells including cell cycle regulation, apoptosis and EMT were downregulated in AKAP4 depleted cells. Also, AKAP4 depletion in ovarian cancer cells revealed reduced tumor growth *in vivo*. Thus, AKAP4 may be a potential novel therapeutic target in ovarian cancer and warrants further studies.

## MATERIALS AND METHODS

### Cell lines

Patients derived cell line A10 (established from papillary cystadenicarcinoma of a 44 year-old female patient) was a kind gift by Dr. Kunle Odunsi, Roswell Park Cancer Institute, Buffalo, NY. Caov-3 (origin: ovary, adenocarcinoma) and SKOV3 (origin: ovary; adenocarcinoma; derived from metastatic site: ascites) were procured from ATCC (American type cell culture collection, Manassas, USA). A10, Caov-3 and SKOV3 were grown in Dulbecco's Modified Eagle Media (DMEM) with 10% Fetal bovine sera (FBS). Cells lines were cultured at 37°C in 5% CO2 incubator. All cell lines were regularly checked for mycoplasma contamination through mycoplasma PCR detection kit (Applied Biological Materials Inc., Richmond, Canada).

### *AKAP4* gene, protein expression and flow cytometric analysis

*AKAP4 gene* expression was validated by reverse transcription polymerase chain reaction (RT- PCR) using *AKAP4* forward primer: 5′- *CAGGATCAGAGGGAGCTTGT* -3′ and *AKAP4* reverse primer: 5′- *TCCAGCTCAGAAGGCAACTT* -3′. For internal loading control *β-actin* was amplified using primer forward : 5′-*ATCTGGCACCACACCT TCTACAATGAGCTGCG*-3′ and *β-actin* reverse: 5′-*CGTCATACTCCTGCTTGCTGATCCACATCTGC*-3′. The PCR product was visualised on 1.5% agarose gel electrophoresis. Flow cytometer analysis was carried out to examine the surface expression of AKAP4 in ovarian cancer cells. Briefly, one million cells were harvested and incubated with primary antibody overnight against AKAP4 followed by fixation in 0.4% para-formaldehyde (PFA). Cells were washed and incubated with anti-rat FITC conjugated (Jackson Immuno Research Laboratories, Inc., Baltimore, USA) secondary antibody for 2h at room temperature. The acquisition and analysis was done using BD FACS Verse and analysis was done using BD FACSuite software (BD Biosciences San Jose USA).

### *Gene* silencing study, reagents, antibodies and Western blot

To study gene silencing, plasmid based shRNA with following sequence (AKAP4: 5′- TCTATGTTCACTT GATCGG-3′ (AKAP4 shRNA1, Clone ID V2LHS-53112); 5′- CAAGCGAACGGGCAATTTA-3′ (AKAP4 shRNA2 Clone ID V2LHS-53113); 5′- TTACCAGAGAAGATA GTCG-3′ (AKAP4 shRNA3 Clone ID V2LHS-53116) and 5′- ATCTCGCTTGGGCGAGAGTAAG-3′ (NC shRNA, RHS4430-99147765) was procured from (Frederick, MD, USA) and used as described earlier [16]. CREB shRNA target1 CREB: 5′- CCGGACCAATCCCTTGAGTTATATA CTCGAGTATATAACTCAAGGGATTGGTTTTTTG3′ (CREB1 shRNA1 NM_004379.2-2002s21c1) and CREB shRNA target2 CREB: 5′- CCGGACATTAGCCC AGGTATCTATGCTCGAGCATAGATACCTGGGCTAAT GTTTTTTG3′ (CREB1 shRNA2 NM_004379.2-284s21c1) was a kind gift from Dr. Subba Rao Gangi Setty, Department of Microbiology & Cell Biology, Indian Institute of Science, CV Raman Ave., Bangalore. Caspase inhibitor (Z-VAD-FMK) was procured from BD Bisciences. Free radical scavenger N-Acetyl-L- cysteine (NAC), Proteosome inhibitor (MG-132) and PKA inhibitor H89 dihydrochloride was purchased from (Sigma-Aldrich Inc), Hydrogen peroxide (H2O2) was purchased from (Merck, USA). Western blot was carried out as described earlier [16] with following antibodies: AKAP4 (ab56551), Apaf1 (ab2000), Akt (9272S), p-Akt (S473), ATM (2873S), p-ATM (ab81292), Bad (sc-8044), Bid (sc-11423), Bcl-2 (B3170), Bcl-xL (B9429), Bak (sc-7873), Bax (B8554), Caspase 7 (sc-81654), Cdk1 (ab18), Cdk2 (ab7954), Cdk4 (sc-23896), Cdk6 (sc-7961), Cycin A2 (ab137769), Cyclin B1 (sc-7393), Cyclin D1(sc-8396), Cyclin E (sc-56310), Cyto-c (sc-56052), CREB (#9197), E-cadherin (ab1416), N-cadherin (ab 76011), NOXA (sc-56169), p- CREB (9191S), PCNA (sc-25280), P-β-Catenin (sc-57535), pH2AX (05-636), PKAc (610981) p21(sc-817), SLUG (ab51772). Western blot was carried out as described earlier [16]. Briefly, protein lysate (10–60 μg/lane) was resolved on 8%–12% sodium dodecylsulphate-poly acrylamide gel electrophoresis (SDS-PAGE), and immunoblots were developed Immobilon western Chemiluminescent HRP substrate (Millipore Corporation, Billerica, MA) as described earlier [16].

### Cellular proliferation and cell viability assay

Cellular proliferation, cell viability, colony forming ability and wound healing assay was carried out in A10 and Caov-3 cells after AKAP4 shRNA treatment as described earlier [16]. Trypan blue cell exclusion assay was performed as follows, cell suspension of shRNA treated ovarian cancer cells and trypan blue (0.4%) dye was made in 1:1 ratio. Cells were counted under light microscope using hemocytometer chamber.

### Cell cycle arrest and TUNEL assay

Cell cycle arrest after shRNA treatment was evaluated by PI staining as described earlier [16]. Briefly, after 48 hr of shRNA treatment cells were washed and fixed in 70% ethanol overnight. Next day cells were treated with PI stain (1 mg/ml) and RNAse solution and kept in dark at 37°C for 30 min before acquisition. Acquisition and analysis was done using BD-FACS CALIBUR (BD Biosciences, California, USA) and BD FACS Verse BD FACSuite software (BD Biosciences San Jose USA). Caspase mediated DNA nicking was analyzed using Apo-BrdU- Red *in-situ* DNA fragmentation assay kit (Biovision, Milpitas, CA, USA) as described earlier [16]. Acquisition and analysis was done using BD FACS Verse BD FACSuite software (BD Biosciences San Jose USA).

### ROS, NAC, H89, Z-VAD-FMK and MG-132 treatment

CellRox Deep Red was used to measure endogenous reactive oxygen species production in A10 and Caov-3 cells after AKAP4 shRNA treatment. Briefly cells were seeded in six well plate and transfection was carried out next day. After 48 h, cells were treated with 100 μL CellRox Deep Red solution and incubated at 37°C for 30 min and acquired at APC-A channel. The results were analyzed using BD FACS Verse and analysis was done using BD FACSuite software (BD Biosciences San Jose USA). PI staining and western blot was carried out in Caov-3 cells at 24 h after H2O2 (40 μm and 80 μm) and H89 (30 μm) treatment. N-acetyl cysteine (NAC) pre-treatment (4 mm for 4 h) was given to cells before transfection to quench ROS generation. Caspase inhibitor (Z-VAD-FMK) was pre-treated (20 μm and 40 μm) to cells for 30 mn before transfection. tMG-132 pre- treatment (500 nm for 1 h) was given before transfection and cell lysate was prepared at 48h to carry out western blotting.

### Xenograft study

Animal studies were carried out after obtaining ethical clearance from Institute animal ethical committee (IEAC). Four week old (*n* = 18) severe combined immuno deficiency (SCID) mice were obtained from institute. Xenograft was established by injecting 2–3 million A10 cells in the intra-peritoneum. Once tumor volume reached 50–100 mm3, two groups were made Group 1: control mice (*n* = 9) were injected NC shRNA intra-tumor, Group 2: experimental mice (*n* = 9) were injected (50 μg) shRNA3 on alternate days. Tumor volume was measured using caliper by the formula V = (W(2) × L)/2. The injection scheduled was attended till 47 days and subsequently animals were sacrificed for further studies.

### Statistical analysis

The statistical analysis was done using SPSS 20.0 statistical software package (SPSS Inc., Chicago, USA) for all *in-vitro* and *in vivo* studies to check the significance of *P* value using student's *t*-test (two tailed).

## SUPPLEMENTARY FIGURES


