# Supplementary material for: Role of A-Kinase anchor protein (AKAP4) in growth and survival of ovarian cancer cells
Source: Oncotarget. 2017 May 24;8(32):53124–36. doi: 10.18632/oncotarget.18163 (PMC5581097; doi:10.18632/oncotarget.18163)
Supplement: Supplementary file 1 [file oncotarget-08-53124-s001.pdf]

Role of A-Kinase anchor protein (AKAP4) in growth and survival of ovarian cancer cells

Supplementary Materials

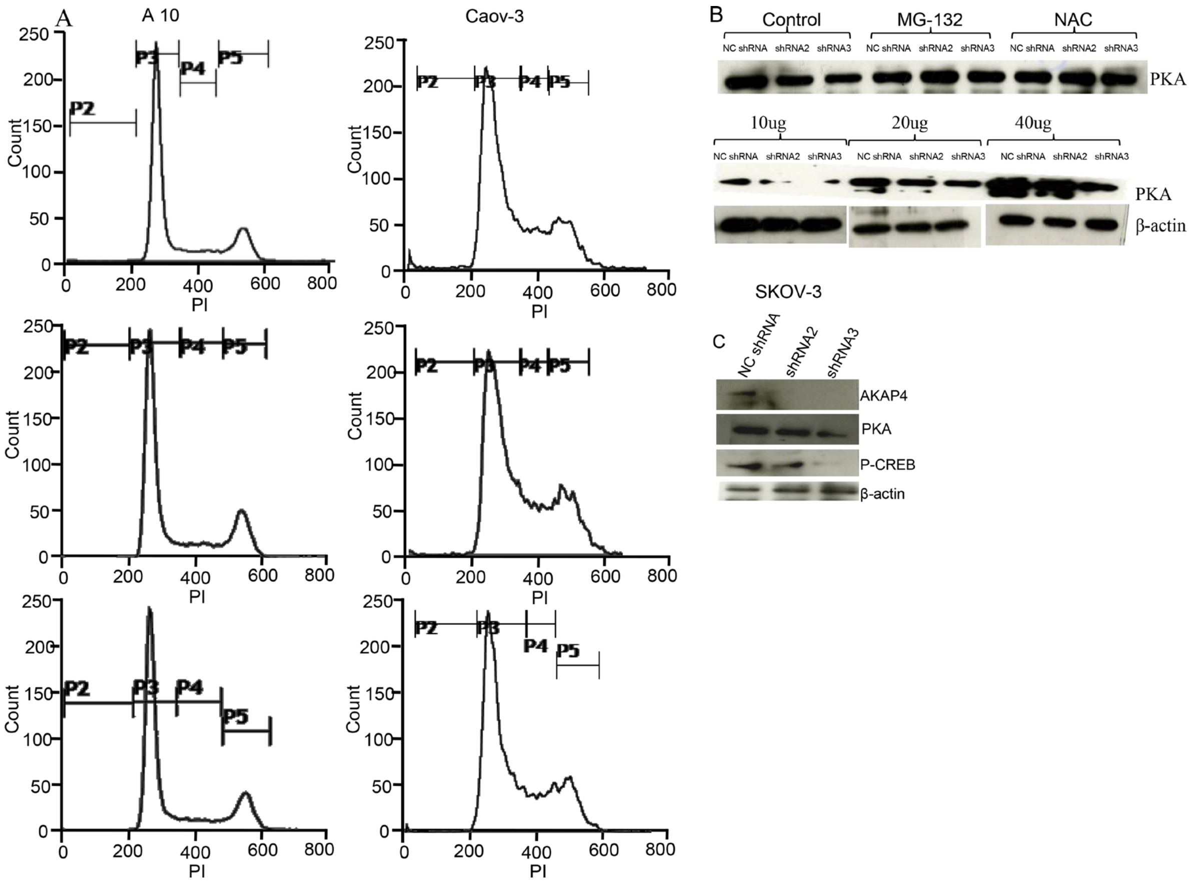

Supplementary Figure 1: (**A**) Histogram analysis after PI staining shows cell cycle rescue after NAC treatment in A10 and Caov-3 cells at 48 hr. (**B**) Western blot shows PKA expression after various treatments (shRNA, shRNA+MG-132 and shRNA+NAC) and PKA expression at different concentration of cell lysate (10 µg, 20 µg and 40 µg) in Caov-3 at 48 hr. (**C**) Western blot shows downregulation of AKAP4, PKA and p-CREB in SKOV-3 cells. β- actin serves as loading control. The data shown as mean ± standard error of the mean (SEM) of three independent experiments. *$P < 0.05$; **$P < 0.01$.

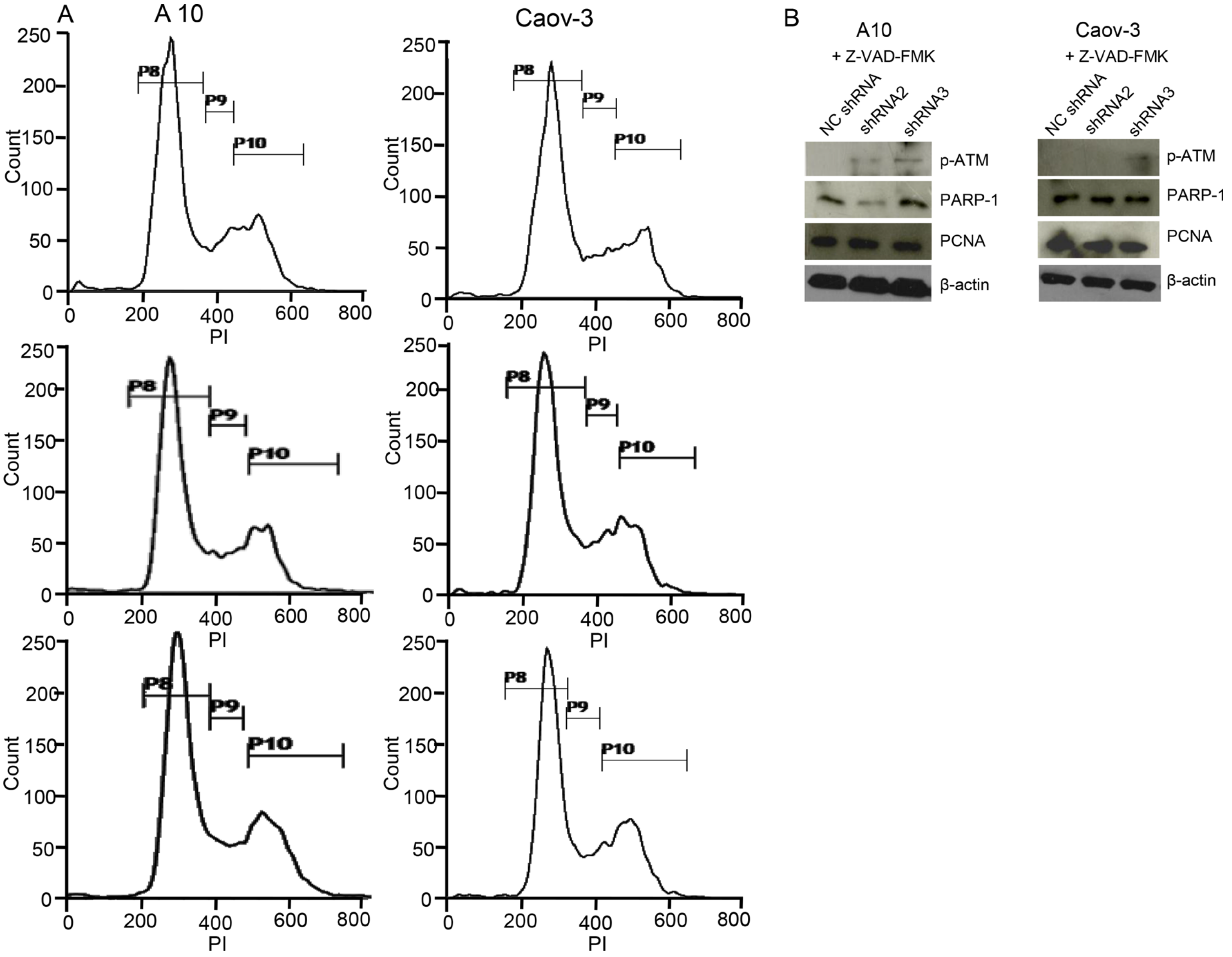

Supplementary Figure 2: (**A**) Histogram analysis after PI staining shows cell cycle rescue after caspase inhibitor (Z-VAD-FMK, 40 μm) pre-treatment in A10 and Caov-3 cells at 48 hr. (**B**) Western blot shows expression of PCNA, PARP1 and p-ATM after caspase inhibition. β-actin serves as loading control.
